# The Impact of Pulsed Electric Fields on Milk’s Macro- and Micronutrient Profile: A Comprehensive Review

**DOI:** 10.3390/foods12112114

**Published:** 2023-05-24

**Authors:** Azizah Mohamad, Nor Nadiah Abdul Karim Shah, Alifdalino Sulaiman, Noranizan Mohd Adzahan, Rai Naveed Arshad, Rana Muhammad Aadil

**Affiliations:** 1 Food Biotechnology Research Centre, Agro-Biotechnology Institute (ABI), National Institutes of Biotechnology Malaysia (NIBM), CO MARDI Headquarters, Serdang 43400, Selangor, Malaysia; 2 Department of Process and Food Engineering, Faculty of Engineering, Universiti Putra Malaysia, Serdang 43400, Selangor, Malaysia; 3 Halal Products Research Institute, Universiti Putra Malaysia, Putra Infoport, Serdang 43400, Selangor, Malaysia; 4 Department of Food Technology, Faculty of Food Science and Technology, Universiti Putra Malaysia, Serdang 43400, Selangor, Malaysia; 5 Institute of High Voltage & High Current, School of Electrical Engineering, Faculty of Engineering, Universiti Teknologi Malaysia, Skudai 81310, Johor, Malaysia; 6 National Institute of Food Science and Technology, University of Agriculture, Faisalabad 38000, Pakistan

**Keywords:** pulsed electric fields, milk, macronutrients, micronutrients

## Abstract

Consumers around the world are attracted to products with beneficial effects on health. The stability, functionality, and integrity of milk constituents are crucial determinants of product quality in the dairy industry. Milk contains macronutrients and micronutrients that aid in a wide range of physiological functions in the human body. Deficiencies of these two types of nutrients can confine growth in children and increase the risk of several diseases in adults. The influence of pulsed electric fields (PEF) on milk has been extensively reviewed, mostly concentrating on the inactivation of microbes and enzymes for preservation purposes. Therefore, the information on the variations of milk macro- and micronutrients treated by PEF has yet to be elucidated and it is imperative as it may affect the functionality, stability, and integrity of the milk and dairy products. In this review, we describe in detail the introduction, types, and components of PEF, the inactivation mechanism of biological cells by PEF, as well as the effects of PEF on macro- and micronutrients in milk. In addition, we also cover the limitations that hinder the commercialization and integration of PEF in the food industry and the future outlook for PEF. The present review consolidates the latest research findings investigating the impact of PEF on the nutritional composition of milk. The assimilation of this valuable information aims to empower both industry professionals and consumers, facilitating a thorough understanding and meticulous assessment of the prospective adoption of PEF as an alternative technique for milk pasteurization.

## 1. Introduction

Milk is an emulsion of lipid globules and a colloidal mixture of protein and mineral aggregates found in carbohydrate suspension (mostly lactose) [1]. It contains essential nutrients for humans, such as proteins, fats, carbohydrates, vitamins, and minerals [2]. Bovine milk consists of water (~87%), macronutrients that are made up of protein (~3.2%), fat (~3.5%), lactose (~4.8%), and micronutrients, which comprise salts, vitamins, and minerals [3]. Macronutrients provide energy to humans and are needed in large amounts [4], while micronutrients are essential elements that are needed in different amounts by humans to perform various physiological functions to maintain health [5]. 

Raw milk is a highly nutritious and safe medium for bacterial growth and must be processed appropriately to reduce risks related to public health. The current industrial practice to maintain the safety and shelf-life stability of milk is pasteurization or sterilization via thermal processing. The Food and Drug Administration has published the Grade “A” Pasteurized Milk Ordinance (PMO) to control the standards and regulations on the production, processing, and marketing of milk and dairy products, and this ordinance comprises the comprehensive directives of sanitary facilities, equipment, and practices to ensure the safety and quality of milk [6]. These measures have been effectively used to combat spoilage and pathogenic microbes in milk as well as other food products.

Milk processing can cause the demolition of the milk fat globule membrane and induce interactions between whey protein and casein with membranes, leading to changes in pH, protein, and lactose content, as well as the destruction of vitamins and enzymes, hydrolysis of proteins and lipids, disruption of calcium and phosphorus equilibrium, and reduction of the cream layer [7,8]. Najib et al. [9] proposed that heat-induced acidification in milk leads to the formation of organic acids, insolubility of tertiary calcium phosphate, and casein dephosphorylation. Borad et al. [10] also reported that thermal pasteurization can lead to dehydration, hydrolyzation, or aggregated milk casein. Sharma et al. [11] found that whey proteins are generally thermolabile due to their globular structure, and thermal processing affects milk functionality. This has led to the exploration of non-thermal processing methods such as pulsed electric fields (PEF), high-pressure processing (HPP), and ultrasound (US) as an alternative to complement or substitute the conventional thermal treatment [12,13].

Pulsed electric fields (PEF) are gaining recognition in food processing due to their energy efficiency, minimal energy loss, flexibility, instantaneity, non-thermal nature, and environmental friendliness [14,15]. It has also been found to reduce spoilage by microorganisms and the inactivation of undesirable enzymes, as well as its better retention of organoleptic and nutritional characteristics [16,17,18]. PEF has great potential in the milk processing industry as it causes little change to the flavour, colour, and nutritional value, and is effective in extending the shelf life of milk [8]. Studies have shown that PEF affects different types of milk including bovine milk [11,19,20,21,22,23,24,25,26,27,28,29], goat milk [12,30], and fruit juice–milk beverages [31,32,33,34]. 

From the literature, it appears that a few researchers have addressed the effects of PEF on milk and its nutrients. With this in mind, we need to get an overview of the mechanisms by which nutritional changes occur, the factors affecting them, and ways to deal with the changes before PEF can be commercially applied in the milk processing industry. Milk macro- and micronutrients are also extremely sensitive to chemical and physical treatment [2]. Therefore, in this review paper, we aim to compare and consolidate all the information on the effect of PEF on milk’s nutrients including fats, fatty acids, proteins, amino acids, vitamins, and minerals. It provides background information for future research areas needed to apply PEF in the dairy and milk processing industries.

## 2. Pulsed Electric Fields System

The PEF system consists of a few components, including a repetitive high-voltage pulse generator, treatment chamber, pump or liquid handling system, and control devices [14,35]. Depending on the design of the treatment chamber and the type of treated sample, either a continuous or batch chamber can be operated. A continuous chamber is preferred for industrial applications due to the continuous flow of the samples, while a batch or static chamber can only process the given amount of liquid, solid, or semi-solid samples [14]. Nevertheless, both chambers can achieve similar output and comparable levels of microbial and enzymatic inactivation [14,36,37]. 

In the PEF process, the sample is positioned in the middle of two or more electrodes before being exposed to high-voltage electric field pulses [38]. PEF can operate at a wide range of ambient temperatures, including ambient (20–25 °C), sub-ambient (<20 °C), and above-ambient (<25 °C) temperatures [39,40], with a short processing time (µs − ms) [18]. PEF can also be applied by varying processing parameters such as electric field strength, pulse width, and pulse frequency, depending on the sample compositions and processing objectives to be acquired [14,18,36,41].

**Figure 1:**
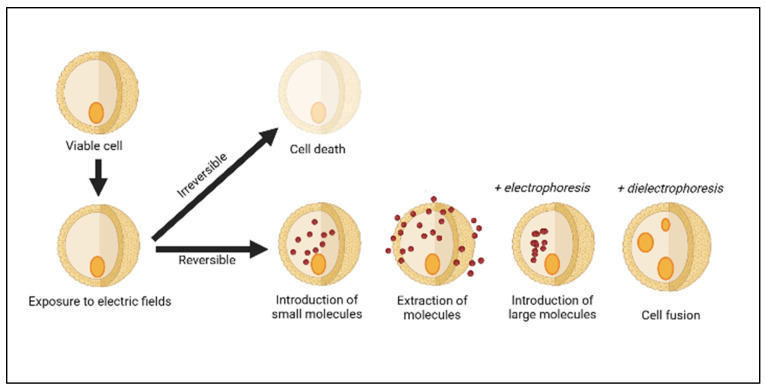
Cell electroporation outcomes are dependent on the pulsing dynamic (amplitude, shape, and duration of pulses) and other cell manipulation approaches (modified and adapted from [42]).

Generally, two mechanisms have been suggested for cell inactivation by PEF, namely electrical breakdown and electroporation [18]. Electrical breakdown occurs when the PEF achieves a greater electric field strength than the critical field strength of the microorganism, resulting in direct discharge and the breaking down of the cell membranes [42]. The biological cells are an electrolyte enclosed by an electrically injured membrane, which is exposed to an extrinsic electric field and subsequently induces transmembrane voltage [38]. The possible outcomes of cell electroporation are shown in Figure 1. Electroporation is the process of temporarily weakening the lipid bilayer and proteins of the cell membranes, resulting in the transformation of cell membrane permeability and the subsequent draining of the membrane [43,44]. It can be reversible or irreversible, depending on the PEF processing parameter, medium constituents, type, and size of the cells [14]. PEF has been used in a variety of food processing applications such as meat processing [45] and the retention of bioactive compounds from fruits and vegetables [46]. Although irreversible, PEF is used for microbial and enzyme inactivation in liquid foods such as milk and fruit juices [30,47].

## 3. Effect of Pulsed Electric Fields on Milk Nutrients

Table 1, Table 2, Table 3 and Table 4 summarize the effects of PEF on fats and fatty acids, proteins and amino acids, vitamins, and minerals in milk, respectively. 

### 3.1. Fats and Fatty Acids

Fats or lipids are the primary elements of milk that influence the cost, nutrition, functional, and sensory attributes of dairy products [54]. A milk fat globule membrane (MFGM) comprises polar lipids, cholesterol, proteins, glycoproteins, and enzymes [22]. The composition and structure of milk fat globules (MFG) and MFGM and their composition contribute to lipases’ access to lipids, which can result in structural changes in the MFGM [19]. Therefore, it is essential to review the effect of PEF processing on the modification of the fatty acid compositions in milk.

The temperature has a significant effect on determining the rate of lipid oxidation since fats and FAs are susceptible to oxidation [55]. Lipid oxidation products are thought to be a risk factor for human health because of their mutagenic, carcinogenic, and cytotoxic characteristics [56]. Temperature influences chemical reactivity, colloidal structure organization, and stability, as well as the rate of synthesis and decomposition of hydroperoxides [57]. Despite the fact that PEF is a non-thermal technique, ohmic heating can cause temperatures to increase [12]. Ohmic or joule heating in the PEF process emerges when an electric current flows through a sample and ions and other charged molecules, such as lipids and proteins, take on the opposite charge of the electrode [56]. 

Earlier, McAuley et al. [22] demonstrated that PEF treatment (30 kV/cm; 22 μs; 2.4 L/min; 53 and 63 °C) had no significant effect on fat, protein, lactose, total solids, and the total solids non-fat of raw whole milk (4% fat). However, the authors observed a minor increase in the milk lipid-derived short-chain FFAs, namely butanoic (C4:0), hexanoic (C6:0), and octanoic (C8:0) acids, during two weeks of refrigerated storage at 4 and 8 °C, due to the greater rate of milk triglyceride hydrolysis by milk-derived lipases and/or esterases (lipoprotein lipase, LPL) and milk microbiota [22]. LPL is the main native milk enzyme. It only becomes active on the fat in natural MFGs when physical treatments have broken them down or if certain blood serum lipoproteins are present [58]. Nevertheless, they propose that the LPL activity is typically less active in milk fat because it is being sheltered by the MFGM and glycoproteins in the skim fraction that inhibit the lipolysis process. Similarly, the changes in FA profiles were also observed by other researchers [30]. They found a significant reduction (*p* < 0.05) in total SFAs and total PUFAs, while the number of total MUFAs increased in PEF-treated goat milk (20, 30, and 40 kV/cm; 5 and 10 μs; 2.5 L/h; 30 °C). The authors suggest that the changes in FA may have been caused by the breakdown of fat and triacylglycerol from MFGM and the catalytic oxidation of heterogeneous metals (Fe and Ni) from the PEF electrode material, which resulted in significant decreases in various FAs. Moreover, some other studies reported that nickel showed the best isomerization properties for FA [59]. According to Ghnimi et al. [57], heterogeneous catalysis by metal oxides can initiate FA oxidation by generating micelles comprising primary hydroperoxide, water, and other amphiphilic compounds. They also suggest that the metal oxide can provide a surface site catalyst for hydrocarbon deprotonation on the nanoscale since the C–H bonds of hydrocarbons are very weakly acidic and unfit for hydrocarbon deprotonation. Despite that, further investigation is needed to understand the PEF electroporation and heterogeneous metal catalysis effects on fatty acid molecules.

According to Yang et al. [19], PEF treatment at 16 kV/cm; 30 µs; 100 mL/min; 25 °C slightly reduced the fat contents and FFA of three types of milk samples: raw milk (RM), milk with small fat globules (MS), and milk with large fat globules. Nevertheless, the same authors found that the MS with the smallest MFGs and the most modified MFGM exhibited a minor increase in FFA following the same PEF treatment. The authors proposed that the changes in FFA are likely due to the electroporation of MFGMs that exposed triglycerides to lipases and subsequently led to the release of FFA. These findings concur well with those of Garcia-Amezquita et al. [60], who found that PEF processing (36 and 42 kV/cm; 2.6 µs; 23 L/h; 25 °C) led to the clustering of small milk-fat globules, which appeared to increase the population of larger globules. However, the actual mean diameter of MFG was unaffected by PEF. It has been reported that PEF processing may result in oxidation (loss) and/or reduction (gain) in both the electrode and the electrolyte (sample) due to electrochemical reactions that took place when the interface voltage exceeded the threshold voltage [30,61]. Therefore, the decrease in fat content can be attributed to the partial loss of some fat clumps that are attached to the PEF tube or electrode. 

### 3.2. Proteins and Amino Acids 

Milk proteins consist of an unstable micellar phase, known as casein, and a soluble phase of whey proteins [54]. The casein phase contains approximately 80% of the proteins and is classified as αs1, αs2, β, and κ-caseins [62]. Whey-phase proteins with the highest concentrations are α-lactalbumin (α-La) and β-lactoglobulin (β-Lg) [63]. Milk contains approximately 3.5 g/100 mL of high-quality protein, comprising nine essential amino acids required by humans [64]. Furthermore, protein components are present in the MFGM as lipoproteins, with casein having the highest content (~80%) of proteinaceous substances in bovine milk, followed by whey protein (~20%); this ratio varies by species [64]. 

Generally, PEF treatment affects the structure of proteins, especially the secondary and tertiary structures [65] leading to denaturation and consequent changes in their functional properties such as solubility, gelation, and aggregation. PEF has been described to affect the structure of proteins and amino acids, which are primarily associated with weaker bonds such as disulfide bonds, hydrogen bonds, and hydrophobic bonds [66]. In addition, disulfide bonds are the main covalent bonds resulting from the PEF-induced protein aggregates [50]. As a result, PEF may promote protein augmentation via hydrophobic and thiol or disulfide reaction aggregation, altering protein thermal stability and enzymatic digestibility [67]. Additionally, it has been demonstrated that some chemical bonds, such as peptides and non-covalent bonds, as well as electrostatic, hydrophobic, and Van der Waals interactions, counterbalance the protein structure [68]. The author also suggests that there is a considerable chance that the application of an electric field may affect the conformation of proteins, due to the presence of electrostatic forces in the peptide chain. These have a good correlation with Yu et al. [53], who found that the alteration of the enzyme by PEF may have reduced the proteolytic enzyme characteristics of milk, resulting in less peptide and amino acid production in PEF-treated milk (30 kV/cm; 80–120 pulses; 6 mL/min; 50 °C). The author also discovered that an increase in pulses may cause an enzyme modification to occur more severely, and that a decrease in enzyme activity may cause a decrease in the production of peptides and free amino acids.

Furthermore, Yang et al. [19] observed that the β-Lg band in milk with small fat globules (MS) was reduced following PEF treatments by 8% (9 kV/cm; 30 µs; 100 mL/min; 25 °C) and 15% (16 kV/cm; 30 µs; 100 mL/min; 25 °C), whereas raw milk (RM) and milk with large fat globules (ML) demonstrated no significant changes in the β-Lg band. Likewise, the band for α-La in MS was similarly reduced following PEF treatments by 11% and 26% at 9 and 16 kV/cm, respectively (30 µs; 100 mL/min; 25 °C); but α-La in RM showed no changes and ML showed just a minor decrease (2–5%). The authors suggest that following PEF treatments, β-Lg and α-La were primarily adsorbed to the small MFGs in MS. They also found that the primary MFGM proteins, xanthine oxidase/dehydrogenase (XO/XDH), were present in the RM sample. The ML sample has a comparable profile for the MFGM proteins; however, the MS sample exhibits a very different profile since it almost completely lacked the XO/XDH band and had additional lower molecular weight protein bands as well as numerous fuzzy protein aggregation bands [19]. The authors also suggest that the MFGM composition of small MFGs differs significantly from that of large MFGs in ML, proving that the membrane of the small MFGs underwent more profound modifications throughout the separation process. 

Other studies have suggested that PEF treatment can alter protein polarity and the microenvironment of amino acid residues due to the liberation of native molecular structures, including the whey protein structure [51]. The changes in the milk protein were also observed by Mathys et al. [24], who found that PEF treatment (40 kV/cm; <15 s; 30 L/h; 20–45 °C) reduced 70% of the native form of milk proteins, including immunoglobulin G (IgG), immunoglobulin A (IgA), and lactoferrin. Moreover, the authors affirm that the variations in milk proteins correspond to the specific energy of PEF treatment. This suggests that high intensity of PEF treatment (>30 kV/cm) along with a mild temperature (40–60 °C) can induce denaturation of milk proteins, possibly due to the unfolding of protein molecules or their positioning towards an applied electric field. These correlate favourably with Sui et al. [52], who perceived significant changes in the concentration of natively folded lactoferrin, aggregated protein, and surface hydrophobicity of whey protein isolate after PEF treatment (30–35 kV/cm, 19.2 and 211 µs; 760 mL/min; 60–70 °C). 

Accordingly, Sharma et al. [69] also reported that the high intensity of PEF could increase the PEF processing temperatures due to higher specific energy, which may lead to the unfolding of protein molecules, the alignment of proteins towards electric fields, and transitions in the structure of the native proteins. They proposed that PEF-induced protein conformational changes are due to denaturation and the formation of intermolecular structures such as β-Lg or κ-casein following cross-linking between casein and serum proteins. In another study, the same authors [21] observed a decrease in MFG size, an increase in ζ-potential, specific surface area, and plasma protein absorption on the MFGM surface of PEF-treated bovine whole milk (20 and 26 kV/cm; 34 μs; 4.2 mL/s; 17–19.4 °C). It has been asserted that the superficial coverage of MFGM through plasma proteins was associated with the increase in ζ-potential, where the ζ-potential is an indication of the degree of damage to the surface of MFGM [70]. Moreover, the decrease in the size of MFG may be attributable to the shear force effect during pumping. These are in agreement with Hemar et al. [28], who reported the shear effect produced during circulating milk through the PEF system that can lead to a partial separation of casein micelles. However, the authors claimed that the effects of higher temperatures (63 °C for 30 min; 73 °C for 15 s) outweighed the modifications brought about by PEF treatment. 

In contrast, Liu et al. [23] found that PEF treatment (49 kV/cm; 19.36 s; 240 mL/min; 20 °C) did not affect the physicochemical properties of milk caseins, the size and ζ-potential of milk particles, the status of the whey protein, or the amount of protein in the serum. The variable medium (sample), processing settings, and PEF system used in the study can be blamed for this rather paradoxical outcome. Nevertheless, these findings appear to be well substantiated by Schottroff et al. [48], who obtained stable immunoglobulin M (IgM) and immunoglobulin G (IgG) in the 10% solution of liquid whey protein treated with PEF (50 kV/cm; 3–8 µs; 5 L/h; 20–40 °C). Since the majority of whey proteins are more susceptible to heat denaturation at neutral pH than under acidic circumstances, they contend that PEF is unlikely to have an impact on soluble proteins. Additionally, the results are consistent with Sharma et al. [20], who discovered that the PEF treatment (20 and 26 kV/cm; 34 s; 4.2 mL/s; 55 °C) had no appreciable impacts on the endothermic peak of bovine whole milk at 32.2 °C. According to this, PEF could maintain protein functioning and integrity by lowering milk protein denaturation, which would lead to less absorption onto the surface of MFGM.

### 3.3. Vitamins

Vitamins are a group of organic complexes that are needed in small quantities; however, a lack of vitamins will lead to such syndromes as scurvy and beriberi. Vitamins fall into two categories: those that are fat-soluble (such as vitamins A, D, E, and K) and others that are water-soluble (such as vitamins B and C). It has been proposed that many variables, including light, temperature, pH, oxygen availability, metal catalysts, and the inclusion of additional antioxidants or reducing agents, can affect the stability of vitamins [68].

According to earlier research, PEF treatment had a negligible impact on milk’s vitamin content [12,33,34,48,71]. Mohamad et al. [12] discovered no significant changes (*p* > 0.05) in vitamin A (as β-carotene) and B-group vitamins (thiamine, riboflavin, and niacin) in PEF-treated goat milk (20–40 kV/cm; 5–13 s; 2.5 L/min; 30 °C). Since vitamin A and B-group vitamins are known to be sensitive to a higher temperature, the higher retention value (>94%) observed in the study is likely attributable to the lower PEF processing temperature (30 °C) used in the study. The authors conclude that extended PEF treatment time and high electric field intensities lead to decreased vitamin retention values. Riener et al. [71] reported that PEF treatment (15–35 kV/cm; 12.5–75 s; 30 °C) had no significant effects (*p* > 0.05) on the levels of thiamin, riboflavin, retinol, and α-tocopherol in fresh bovine raw milk. The effect of PEF (18.3–27.1 kV/cm; 400 μs; 20–25 °C) on both water-soluble (thiamin, riboflavin, and ascorbic acid) and fat-soluble (cholecalciferol and tocopherol) vitamins in milk has been evaluated by Bendicho et al. [72]. The authors observed no significant changes (*p* > 0.05) in both classes of vitamins except for ascorbic acid. Likewise, the reduction of ascorbic acid was found to be dependent on the electric field strength, treatment time, and temperature. However, they argued that PEF provides better retention of ascorbic acid (vitamin C) compared to thermal treatment (63 and 75 °C; 15 s and 30 min). 

PEF treatment has also been demonstrated to effectively maintain the bioaccessibility of provitamin A (carotenoids) from fruit juices and milk beverages. For instance, Rodríguez-Roque et al. [32] observed that PEF treatment (35 kV/cm; 1800 s; 60 mL/min; 35 °C) increased the bioaccessibility of total carotenoids in fruit juices (orange, pineapple, kiwi, and mango) and milk beverages by 15%. They found that PEF treatment gives greater stability to the total carotenoids due to the inactivation of oxidative enzymes, the interruption of cell membranes and proteins, and the release of some individual carotenoids. These are consistent with Zulueta et al. [33], who obtained minimal losses of ascorbic acid and total carotenoids with only 2 and 5% destruction rates, respectively, in PEF-treated orange juice-milk beverages (25 kV/cm; 280 µs; 60 mL/min; 57 °C). Salvia-Trujillo et al. [34] also observed higher retention (95–99%) of group B vitamins (niacin, thiamin, and riboflavin) in PEF-treated fruit juice–whole milk (FJ–WM) and fruit juice–skim milk (FJ–SM) beverages (35 kV/cm; 1800 µs; 760 mL/min; <40 °C). Following this, they discovered increased vitamin C retention (>97%) in PEF-treated FJ–WM and FJ–SM beverages, as opposed to 50% retention following 21 days of storage at 4 °C. The results demonstrate that water-soluble vitamins, particularly niacin, thiamin, and riboflavin, were not oxidatively degraded by PEF. They demonstrate that water-soluble vitamins are more responsive to heat treatment than to PEF treatment by contrasting heat (90 °C) and PEF treatment (40 °C). These findings are in good agreement with those of Schottroff et al. [48], who reported that after PEF treatment (50 kV/cm; 3–8 s; 5 L/h; 20–40 °C), there were no significant losses (*p* > 0.05) of vitamins A and C in liquid whey protein. The findings would seem to indicate that PEF treatment does not subject vitamins to any chemical changes.

### 3.4. Minerals

Milk contains 20 minerals that are necessary for human nutrition. The two groups of these minerals are called macro- and microminerals. These macro- and microminerals occur in milk in an equilibrium between the soluble and colloidal phases, each serving a different physiological purpose [73]. For instance, Na and K are crucial in preserving the osmotic equilibrium between blood and milk, while Mg, Ca, P, and Zn are attached to casein micelles in milk, making their content directly tied to casein content [74]. It has been reported that Mg varies by species and is frequently connected to milk protein [75]. Although milk only contains 8–9 g/L of minerals, it is nevertheless an important source of nutrition [76].

The impact of PEF on the mineral profiles of milk has not yet been extensively researched. Our literature search revealed that there is still some pertinent research on the effects of PEF on milk minerals in other species of animals. For instance, Mohamad et al. [31] found that the Fe levels in goat milk treated with PEF increased significantly (*p* < 0.05) at 20–40 kV/cm, 5–10 s, 2.5 L/h, and 30 °C. However, after PEF treatment, it was determined that the other minerals (Cr, Ni, and Mn) were not significant (*p* > 0.05). The authors concluded that the electrochemical reaction that takes place during PEF treatment may have caused the metal components from the PEF electrode to dissolve into the sample. The results obtained are in close agreement with those of Gad and Jayaram [25], who investigated the release of metal particles from stainless-steel electrodes in samples of 2% partially skimmed milk. The Fe concentration rose by 500 µg/L following PEF treatment (30–40 kV/cm; 500 mL/min; 20–25 °C) as opposed to 300 µg/L in untreated samples. The authors agreed that the commercialization of PEF processing may be impacted by electrochemical reactions that happen during milk processing about safety and quality. 

## 4. Current Challenge and Future Outlook

PEF is an innovative non-thermal processing technique with vast potential for preserving food products, including milk. While PEF technology is heading towards wider industrial applications, there are still some limitations that hinder the commercialization and integration of PEF in the food industry. Despite the many positive aspects of PEF technology in milk processing that have been reported, the application of PEF in the dairy industry is still fairly limited. Several factors such as high capital investment, changes in the conventional layouts of the milk processing plant, and other requirements to optimize PEF conditions for specific product applications have prevented the industry from investing in PEF equipment. Besides that, issues related to the regulatory aspects, toxicity risks, and consumer acceptance need to be addressed, as they can affect the commercialization of PEF through equipment reliability, safety, and quality aspects. The development of a PEF system with a reliable yet affordable, large-scale generation of high-voltage electric fields is indeed not a straightforward task. Hence, to meet the industrial demands and targets, a high-voltage power supply is the main focus that should be emphasized. Concerning safety and quality issues, electrochemical reactions that take place during PEF processing may influence the commercialization of the PEF system. These electrochemical reactions that arise from the electrode–electrolyte (food sample) interfaces need to be minimized in order to reduce electrode corrosion and migration of electrode material into food products, resulting in the corrosion of the electrodes, electrolysis of water, and migration of electrode materials into the food. Nevertheless, the intensity of the electrochemical reaction can be minimized by reducing pulse parameters, including pulse width and frequency, and by the application of durable electrode materials such as carbon as well as a chemically resistive electrode such as chromium, titanium, and platinum.

PEF treatment alone cannot destroy vegetative bacteria, which makes it impossible to eliminate the spores. Even at the high intensity of electric fields, bacterial spores or ascospores in food products are commonly resistant to PEF. Likewise, PEF cannot inactivate enzymes, and products need to be stored at low temperatures to control enzymatic reactions. In short, PEF-treated products are not shelf-stable because they are pasteurized and require refrigeration. Therefore, hurdle technologies are available to improve overall PEF processing. Additionally, a combination with mild heat could be an improvement to sole PEF processing. Besides that, PEF is inapplicable for liquids containing particulates or gases and foods with high electrical conductivity without a pre-treatment process. 

The effect of PEF on milk nutrients makes it important to explore the use of PEF as an alternative preservation technology to improve the functionality of milk. To understand the mechanisms of modification by PEF in the macro- and micronutrients of milk, more fundamental studies are needed. Over the past few years, research efforts on milk have focused on the inactivation of microorganisms and enzymes. Only a few studies mentioned the nutritional content, including macro- and micronutrients, of milk. Moreover, the data published so far are not well grounded and quite inconsistent, possibly due to the difference in the PEF models, processing parameters, and medium composition. However, the effects of PEF technology seem promising for research on fruits and vegetables.

In the future, perhaps we will foresee extensive research on the impact of PEF on milk bioaccessibility. Specific studies on the materials science of milk constituents may anticipate the disruption and restructuring of building blocks in the milk matrix. Hence, the wholesomeness of the physicochemical, biological, functional, and sensory properties of milk and dairy products will be assured. The study was also able to explore the modification of molecules, including the macro- and microstructures of milk nutrients. In addition, the development of cutting-edge in situ methods may be required to elucidate the modifications of macro- and micromolecules in milk nutrients.

## 5. Conclusions

In conclusion, this review outlines that PEF treatment may cause minor changes to macro- and micronutrients, including fats, fatty acids, proteins, amino acids, vitamins, and minerals in milk. Nevertheless, PEF can preserve the macro- and micronutrients in milk. Taken as a whole, it is important to study the effect of PEF on milk nutrients because these constituents are more susceptible to degradation and may react with other components, which subsequently may affect the biomolecules’ structural, functional, and sensorial properties. We proposed that further research on material sciences should be undertaken to provide a clear understanding of the effects of PEF on macro- and micronutrients in milk. Although PEF exhibits a minor reduction in protein and fat content, the results published so far appear to be ill-defined and debatable. Therefore, further research is needed to find out how PEF affects milk nutrients. On a wider level, more in-depth research is also needed to determine the mechanisms and factors that contribute to the impact of PEF on variations in macro- and micronutrients in milk. To sum up, PEF has great potential to be commercially applied in the milk processing industry, coupled with the advantages of being energy-efficient and environmentally friendly. Despite that, a few constraints have delayed its incorporation into a commercial application. One of the limitations of PEF is associated with the electrochemical reactions that arise from the electrode-electrolyte (food sample) interfaces and result in the corrosion of the electrodes, electrolysis of water, and migration of electrode materials into the food. Nevertheless, the intensity of the electrochemical reaction can be minimized by reducing pulse parameters, including pulse width and frequency, and by applying durable and chemically resistive electrode materials such as carbon, chromium, titanium, and platinum. Because of the complexity of milk, we recommend combining PEF with different hurdle processing methods to increase PEF efficacy. Combining PEF processing with mild heating (<55 °C), for example, can achieve microbial stability and milk safety at the lowest PEF intensity while reducing the effect of electrochemical reactions. 

## Figures and Tables

**Table 1:** Effects of PEF on the fats and fatty acids in milk.

Nutrient Constituents	Products	PEF Parameter	Effects	References
Fats and fatty acids	Raw bovine milk and milk with different MFG sizes	16 kV/cm; 30 µs; 100 mL/min; 25 °C	Increase in FAs and some unknown long-chain FAs in raw milk and milk with large MFGs; a slight decrease in the fat content	[19]
Fatty acid profiles	Raw goat milk	20, 30, and 40 kV/cm; 5 and 10 μs; 2.5 L/h; 30 °C	Reduction in the total SFAs and total PUFAs; increase in total MUFAs	[30]
Fatty acids	Fruit juices-milk beverages	35 kV/cm; 1800 μs; 60 mL/min; 60 mL/s; <40 °C	20% increase in linoleic acid; decrease in palmitic, linoleic, and linolenic acids (12–20%) at day 56	[31]
Fatty acid profiles	Raw whole bovine milk (4% fat)	30 kV/cm; 22 μs; 2.4 L/min; 53 and 63 °C	No significant changes (*p* > 0.05) in fat content; minor increase in butanoic (C4:0), hexanoic (C6:0), and octanoic (C8:0)	[22]
Fats	Bovine whole milk	20 and 26 kV/cm; 34 μs; 4.2 mL/s; 55 °C	Increase fat melting from a-crystal into b-crystal conversion	[20]
MFG	Bovine whole milk (44% fat)	20 and 26 kV/cm; 34 μs; 4.2 mL/s; 17–22 °C	Reduction in MFGs’ size; increase in ζ-potential and specific surface area; plasma protein absorption on MFGM surface	[21]
Milk fat	Reconstituted skimmed milk	15–20 kV/cm; 20–60 pulses; <35 °C	Increase the surface area of MFGs	[27]
Volatile compounds (including fatty acids)	Raw bovine milk	15–30 kV/cm; 800 µs; <40 °C	No significant changes (*p* > 0.05) in methyl ketones [saturated fatty acids); no changes in concentration of short-chain fatty acid	[29]

Milk fat globule membrane (MFGM), fatty acid (FA), saturated fatty acid (SFA), monounsaturated fatty acid (MUFA), polyunsaturated fatty acid (PUFA).

**Table 2:** Effects of PEF on the milk proteins and amino acids.

Nutrient Constituents	Products	PEF Parameter	Effects	References
Proteins	Raw bovine milk and milk with different MFG sizes	16 kV/cm; 30 µs; 100 mL/min; 25 °C	15% and 29% decrease in β-Lg and α-La respectively (in milk with small MFGs); more small protein aggregates (<40 kDa) appeared	[19]
Native proteins	10% solution of liquid whey protein	50 kV/cm; 3–8 µs; 5 L/h; 20–40 °C	Stable IgM and IgG	[48]
Whey proteins	Bovine whole milk	20 and 26 kV/cm; 34 μs; 4.2 mL/s; 55 °C	Increase in the hydrophobic surface of the protein; reducion in milk protein denaturation	[20]
Caseins and whey proteins	Reconstituted skim milk (10 wt%)	49 kV/cm; 19.36 µs; 240 mL/min; 20–72 °C	Increase in the amount of protein in the serum; increase the number of caseins in serum; no changes in whey proteins	[23]
Protein compositions	Fresh raw cream and pasteurized cream (40% fat)	37 kV/cm; 1705 μs; 25 mL/min; 50 and 65 °C	No changes in phospholipids; induced interactions of β-Lg and MFGM proteins (at 65 °C).	[49]
Milk proteins	Raw whole milk	40 kV/cm; <15 s; 30 L/h; 72 °C	70% decrease in the native form of milk proteins including IgG, IgA, and lactoferrin	[24]
Protein	BSA	20–35 kV/cm; 400 μs; 200 Hz; <30 °C	No self-aggregation of BSA; no significant difference (*p* > 0.05) in molecular weight distribution profiles of BSA	[50]
Protein content	Raw skim and raw whole milk	30.76–53.84 kV/cm; 12–30 pulses, 1 L/min; 20–40 °C	A 0.17% decrease in protein content	[26]
Whey protein	Whey protein isolate	12–20 kV/cm; 10–20 pulses; <35 °C	Increase polarity and unfolding whey proteins; partial denaturation of whey fractions	[51]
Protein aggregation	Whey protein isolate	30–35 kV/cm; 19.2–211 µs; 60 mL/min; 30–75 °C	No changes in protein aggregation and surface hydrophobicity; partial denaturation of native lactoferrin, apo-lactoferrin, and halo-lactoferrin	[52]
Milk casein micelles’ sizes	Raw milk, reconstituted skim milk, concentrated skim milk, and milk protein concentrates	45 kV/cm; 20 µs; 240 mL/min; 30 °C	Decrease in casein micelles’ sizes	[28]
Proteolysis profiles (peptide and free amino acid concentration)	Raw milk cheese	30 kV/cm; 80–120 pulses; 6 mL/min; 50 °C).	Intermediate proteolysis profiles between raw milk and thermally pasteurized milk Severe enzyme modification reduces the formation of peptides and amino acids at longer pulses	[53]

Milk fat globule membrane (MFGM), bovine serum albumin (BSA), β-lactoglobulin (β-Lg), α-lactalbumin (α-La), immunoglobulin M (IgM), immunoglobulin G (IgG).

**Table 3:** Effects of PEF on the milk vitamins.

Nutrient Constituents	Products	PEF Parameter	Effects	References
Vitamin A (as β-carotene) and B-group vitamins (thiamine, riboflavin, and niacin)	Raw goat milk	20, 30, and 40 kV/cm; 5–13 µs; 2.5 L/min; 30 °C	>94% retention of vitamin A (as β-carotene); 95–99% retention of B-group vitamins	[12]
Vitamins A and C	10% solution of liquid whey protein	50 kV/cm; 3–8 µs; 5 L/h; 20–40 °C	No changes in vitamins A and C	[48]
Total carotenoids (provitamin A), vitamin C, in vitro bioaccessibility of vitamin C	Fruit juice and milk beverages	35 kV/cm; 1800 µs; 60 mL/min; <35 °C	15% increase in the bioaccessibility of total carotenoids (provitamin A); 8–15% reduction of vitamin C; no changes in bio-accessibility of vitamin C	[32]
Total carotenoids (provitamin A), vitamin C (ascorbic acid)	Orange juice-milk beverage	25 kV/cm; 280 µs; 60 mL/min; 57 °C	2% destruction of ascorbic acid;5% destruction of total carotenoids	[33]
B-group vitamins (niacin, thiamin, and riboflavin), vitamin C	FJ-WM and FJ-SM beverages	35 kV/cm; 1800 µs; 760 mL/min; <40 °C	95–99.8% retention of group B vitamins (niacin, thiamin, and riboflavin); 97% retention of vitamin C in FJ-WM; 99.5% retention of vitamin C in FJ-SM	[34]

Fruit juice–whole milk (FJ–WM), fruit juice–skim milk (FJ–SM).

**Table 4:** Effects of PEF on the milk minerals.

Nutrient Constituents	Products	PEF Parameter	Effects	References
Fe, Cr, Ni, and Mn	Raw goat milk	20, 30, and 40 kV/cm; 5 and 10 μs; 2.5 L/h; 30 °C	Significant increase (*p* < 0.05) in Fe; A non-significant increase in (*p* > 0.05) in Cr, Ni, and Mn	[30]
Fe, Cr, and Ni	2% partially skimmed milk	30–40 kV/cm; 500 mL/min; 20–25 °C	A significant increase (*p* < 0.05) in Fe	[25]

## Data Availability

Not applicable.
